# Silver Nanoparticle Effects on Antioxidant Response in Tobacco Are Modulated by Surface Coating

**DOI:** 10.3390/plants11182402

**Published:** 2022-09-15

**Authors:** Karla Košpić, Renata Biba, Petra Peharec Štefanić, Petra Cvjetko, Mirta Tkalec, Biljana Balen

**Affiliations:** Department of Biology, Faculty of Science, University of Zagreb, Horvatovac 102a, 10000 Zagreb, Croatia

**Keywords:** antioxidant enzymes, Comet assay, *Nicotiana tabacum* L., nonenzymatic antioxidants, oxidative stress, ROS, surface coatings, silver nanoparticles, silver ions

## Abstract

The antimicrobial properties of silver and enhanced reactivity when applied in a nanoparticle form (AgNPs) led to their growing utilization in industry and various consumer products, which raises concerns about their environmental impact. Since AgNPs are prone to transformation, surface coatings are added to enhance their stability. AgNP phytotoxicity has been mainly attributed to the excess generation of reactive oxygen species (ROS), leading to the induction of oxidative stress. Herein, in vitro-grown tobacco (*Nicotiana tabacum*) plants were exposed to AgNPs stabilized with either polyvinylpyrrolidone (PVP) or cetyltrimethylammonium bromide (CTAB) as well as to ionic silver (AgNO_3_), applied in the same concentrations, either alone or in combination with cysteine, a strong silver ligand. The results show a higher accumulation of Ag in roots and leaves after exposure to AgNPs compared to AgNO_3_. This was correlated with a predominantly higher impact of nanoparticle than ionic silver form on parameters of oxidative stress, although no severe damage to important biomolecules was observed. Nevertheless, all types of treatments caused mobilization of antioxidant machinery, especially in leaves, although surface coatings modulated the activation of its specific components. Most effects induced by AgNPs or AgNO_3_ were alleviated with addition of cysteine.

## 1. Introduction

Metal nanoparticles (NPs) have unique physical, chemical, and biological traits that are specifically linked to their shape, size, and composition, which is why technologies based on NPs have been exploited in the fabrication of a constantly growing number of commercial products [1,2]. Consequently, NPs are being released into aquatic and terrestrial systems via a number of pathways, which raises apprehension over their possible detrimental effects on living organisms [3]. Among different available metal NPs, silver nanoparticles (AgNPs) are the most frequently applied ones because of the well-known antibacterial and antifungal properties of silver, due to which AgNPs have been utilized in a wide range of medical and healthcare products [4] as well as in agriculture as nanopesticides and nanofertilizers [5]. Therefore, it is of crucial importance to intensively study their possible toxicity following various modes of exposure [6]. Studies performed so far have shown that AgNPs can induce toxicity in bacteria [7,8], freshwater and marine algae [2,9], aquatic and terrestrial animals [10,11,12,13]), and plants [14,15,16,17] as well as in human cell lines [18,19]. The toxicity of AgNPs has often been attributed to the generation of reactive oxygen species (ROS) [18], which induces disruption of the cell membrane [7] and damage of protein or DNA molecules [20]. However, it is still not clear to which degree AgNP toxicity results from nanoparticulate form and how much toxicity is related to the released silver ions (Ag^+^) [6].

Plants are primary producers in any ecosystem and as such have a crucial role in the accumulation and biodistribution of many substances delivered into the environment. It can be expected that they will interact with AgNPs, thus serving as a potential route for their transport and bioaccumulation into food chains [21]. Plants can be affected by AgNPs either directly, through the application of the commercially available products that are being implemented in agriculture, or indirectly, via AgNP-containing products for human usage that are being released to the environment [20]. Regardless of the way of exposure, studies have shown that AgNPs can induce toxic effects in plants, which is related to their uptake, distribution, and translocation within the plant [22], although the exact mechanism on how they affect plant growth has not yet been fully elucidated. Studies have shown that upon uptake, AgNPs can be accumulated and deposited in the plant cell wall, intermembrane space, and within the cell [14,15,23]. Changes in the germination rate, fresh and dry weight, and root and shoot length are frequently observed markers of the AgNP-induced phytotoxicity [2,20,24]. AgNP toxic effects have also been confirmed at the physiological level based on a reduction of chlorophyll synthesis and nutrient uptake, decline of transpiration rate, alteration of hormone levels, and impaired photosynthetic performance [25,26]. All these effects implicate toxicity on the cellular level. Indeed, plant cell toxicity studies have undoubtedly proven that AgNPs can increase ROS production and therefore exert damage to important biomolecules (such as lipids, proteins, and nucleic acids) as well as provoking changes in the activity of plant hormones or antioxidant enzymes [14,15,23,27,28,29], which suggests that oxidative stress could have an important role in the phytotoxicity of AgNPs. Several studies have also shown that AgNPs can induce cyto- and genotoxic effects in plants [14,30], which indicate their ability to adversely damage different cell structures, affect the rate of cell division, induce genomic instability, or cause irreparable cell death [31].

It is well documented that applied plant model and its developmental stage as well as experimental setup, such as AgNP dosage, exposure duration, and medium composition, greatly determine AgNP-induced effects [20,24] However, physicochemical characteristics of AgNPs themselves (coating, surface charge, and particle size) also substantially contribute to the potential phytotoxicity [25,32,33,34], particularly since AgNP stability and susceptibility to transformation upon synthesis are directly related to these AgNP attributes [35]. Bioavailability and biological effects of AgNPs are usually affected by the processes of agglomeration and aggregation, which result in the formation of larger particles, oxidation of elemental silver (Ag^0^) to silver ion (Ag^+^), and subsequent dissolution to dissolved Ag^+^ species, thus modifying AgNP reactivity [36]. The most common way to ensure AgNP stability is the addition of various surface coatings during their synthesis, and for that purpose, different polymers (polyvinylpyrrolidone, PVP and polyethylene glycol, PEG), surfactants (cetyltrimethylammonium bromide, CTAB and sodium dodecyl sulphate, SDS), polysaccharides (gum arabic, GA), and carboxylic acids (citrate) can be applied [24]. However, coating agents can impact AgNP solubility and reactivity [24,37] and thus affect their behavior and transformations in the exposure medium [38], consequently influencing phytotoxic effects [14,25,34]. Although there are numerous research papers that investigate the phytotoxic effects of AgNPs stabilized with various stabilizing coatings, only a few studies compared the effects of differently coated AgNPs in the same experiment (reviewed in [24]) and mostly found that differently coated AgNPs induce differential plant response.

In our previous paper, we presented the results on the stability of differently coated AgNPs (citrate, PVP, and CTAB) and AgNO_3_ in the plant culture medium and their effects on photosynthesis of tobacco plants [25]. AgNP–citrate exhibited the least impact on pigment content and photosynthesis performance, which was correlated with their rapid agglomeration in the exposure medium and consequently weak uptake. On the contrary, AgNP–PVP and AgNP–CTAB induced a deterioration of photosynthetic activity and pigment reduction as well as alterations in chloroplast ultrastructure, which has been ascribed to their higher stability, elevated Ag accumulation, and surface charge. Therefore, in the current study, we aimed to investigate the impact of AgNP–PVP and AgNP–CTAB on the appearance of oxidative stress and antioxidant response in tobacco plants in the same experimental conditions in order to reveal if intrinsic properties of differently coated AgNPs affect the antioxidant status of tobacco plants as well. Moreover, this study also included exposure to AgNO_3_ treatments as well as combined treatments of all silver forms with cysteine, a strong ligand of Ag^+^ ions [39], in order to investigate if the impact on ROS formation and oxidative stress is dependent on the form of Ag.

## 2. Results

### 2.1. AgNP Characterization

Both types of AgNPs investigated in this study were characterized using UV-Vis spectroscopy, transmission electron microscopy (TEM), dynamic light scattering (DLS), and electrophoretic light scattering (ELS), and the results are presented in Table S1. UV-Vis spectra showed surface plasmon resonance (SPR) peaks of 465 nm for AgNP–PVP and 410 nm for AgNP–CTAB, thus confirming the synthesis of AgNPs of 80 and 40 nm nominal diameters, respectively, as previously reported in Peharec Štefanić et al. [25]. The quantity of ionic Ag in the synthesized dispersions was ≤0.5% (Table S1). DLS-determined volume size distributions indicated hydrodynamic diameters (dH) of 58 nm and 56 nm for AgNP–PVP and AgNP–CTAB, respectively. A peak corresponding to dH of about 28 nm, noted for AgNP–CTAB, was ascribed to small particles arising at the beginning of the reaction from rapid Ag reduction when the ratio of reducing agent to Ag was high. Small agglomerates were evidenced by a population with a dH of about 162 nm. The zeta (ζ) potential values were found to be −4 mV for AgNP–PVP and 45 mV for AgNP–CTAB (Table S1), as previously reported in Peharec Štefanić et al. [25].

### 2.2. Ag Content

#### 2.2.1. Root

In root tissue, silver uptake exhibited a dose-dependent effect in all tested treatments with either AgNPs or AgNO_3_. The uptake was significantly higher in plants exposed to both types of AgNPs than AgNO_3_ at all tested concentrations. In all combined treatments with cysteine, the Ag accumulation was significantly reduced compared to corresponding treatments without cysteine, which was particularly pronounced in treatments with ionic silver; however, the addition of cysteine was not able to completely abolish Ag uptake (Table 1).

#### 2.2.2. Leaf

Silver accumulation in leaf showed a dose-dependent response in all treatments with either AgNPs or AgNO_3_ applied alone. Cysteine addition substantially decreased Ag uptake in all combined treatments compared to the exposure with a corresponding treatment with AgNPs or AgNO_3_ alone. In general, Ag accumulated more in leaves of plants exposed to both types of AgNPs than in those treated with AgNO_3_ at all tested concentrations (Table 1).

### 2.3. Oxidative Stress Parameters

#### 2.3.1. Root

All AgNP–PVP treatments induced ROS formation in comparison to the control, although only the 25 µM concentration resulted in a significantly elevated value, which was successfully alleviated with cysteine addition. None of the AgNP–CTAB treatments resulted in significant changes in ROS content compared to the control, while cysteine addition had no effect in the combined treatments. Among AgNO_3_ treatments, only the 25 µM concentration resulted in a significantly elevated value in comparison to the control; however, it was significantly reduced upon the addition of cysteine. A comparison of the values obtained for each concentration showed that AgNP–PVP at 50 µM concentration induced higher ROS formation than AgNO_3_, while treatment with 100 µM AgNP–PVP resulted in elevated ROS compared to AgNP–CTAB and AgNO_3_ (Table 2).

Malondialdehyde (MDA) content was significantly decreased upon exposure to the highest AgNP–PVP concentration in comparison to the control and treatments with lower concentrations; in the combined treatment with cysteine, a value similar to the control was obtained. None of the AgNP–CTAB treatments had any significant effect on the MDA content, while all AgNO_3_ concentrations resulted in significantly lowered values compared to the control. In combined treatments, cysteine addition resulted in similar or even lower values at 25 and 50 µM AgNO_3_ concentrations. At the lowest tested concentration, both types of AgNPs elevated MDA content compared to AgNO_3_, while at the highest concentration, plants treated with AgNP–CTAB had the highest MDA value (Table 2).

None of the tested treatments with either AgNPs or AgNO_3_ applied alone had any significant effect on protein carbonyl content in comparison to the control. Interestingly, cysteine addition exhibited an impact on the combined treatments with AgNO_3_, where elevated values were recorded in comparison either to the control or to the corresponding concentration of AgNO_3_ without cysteine. A comparison between treatments of the same concentration showed that at two higher concentrations, plants treated with AgNP–PVP had a higher protein carbonyl content compared to those exposed to AgNP–CTAB and AgNO_3_ (Table 2).

None of the investigated AgNP–PVP concentrations induced a significant effect on tail DNA compared to the control; however, the 100 µM treatment resulted in significantly higher values than 25 µM AgNP–PVP. Cysteine addition had no significant effect on DNA damage in any of the combined treatments compared to corresponding concentrations without cysteine. Among treatments with AgNP–CTAB, no increase in tail DNA was observed; at the highest tested concentration (100 µM), even a lower value was recorded in comparison to both the control and other treatments with AgNP–CTAB. The addition of cysteine in the combined treatment resulted in values similar to the control. AgNO_3_, applied in any of the tested concentrations, failed to exhibit any significant difference in comparison to the control, although exposure to the 100 µM concentration resulted in significantly elevated values compared to treatments with 25 and 50 µM AgNO_3_. The addition of cysteine substantially increased tail DNA upon exposure to combined treatments with 25 and 50 µM AgNO_3_, although the obtained values were not significantly different in comparison to the control. A comparison between each type of Ag treatment showed that 100 µM AgNP–PVP and AgNO_3_ induced a higher tail DNA than AgNP–CTAB of the same concentration (Table 2).

#### 2.3.2. Leaf

Among treatments with AgNP–PVP, none of the applied concentrations induced a significant change in ROS formation compared to the control, although the values obtained with the 100 µM AgNP–PVP were significantly higher to those measured upon exposure to 25 µM AgNP–PVP. Cysteine addition resulted in a significantly elevated value only in the combined treatment with 25 µM AgNP–PVP, although it was not significantly different compared to the control. None of the treatments with AgNP–CTAB resulted in significant changes in ROS either in comparison to the control or between different concentrations; the addition of cysteine had no effect on any of the treatments. Two lower AgNO_3_ concentrations (25 and 50 µM) significantly lowered ROS formation in comparison to the control, while exposure to the 100 µM AgNO_3_ resulted in elevated values. Cysteine addition resulted in a significant change in combination with 25 µM AgNO_3_ in comparison to the corresponding treatment without cysteine (Table 3).

All of the treatments with Ag, applied either in the form of NPs or ionic Ag, elevated MDA content in comparison to the control, although none of the values were found to be statistically significant. The addition of cysteine had no effect on MDA content in the majority of the combined treatments; the exceptions were combined exposures with 25 µM AgNP–PVP and 50 µM AgNP–CTAB, in which significantly lower values, similar to control, were obtained (Table 3).

Upon exposure to all Ag treatments, no significant change in protein carbonyl content was recorded compared to the control, although all treatments with AgNP–CTAB induced significantly elevated values compared to AgNP–PVP and AgNO_3_ of corresponding concentrations. Moreover, all combined treatments with AgNP–CTAB and cysteine resulted in reduced values compared to treatments with AgNP–CTAB alone, although significantly only at the lowest concentration (Table 3).

The majority of the tested AgNP and AgNO_3_ treatments failed to induce significant effects on DNA damage compared to the control; the only exception was the exposure to 25 µM AgNP–PVP upon which an elevated value was recorded. Combined treatments with cysteine mostly had no impact on tail DNA length in treatments with AgNPs, while increased values were obtained in all combined treatments with AgNO_3_. A comparison of different Ag treatments revealed that 25 µM AgNP–PVP and AgNP–CTAB had a higher impact than AgNO_3_ of the same concentration (Table 3).

### 2.4. Nonenzymatic Antioxidants

#### 2.4.1. Root

None of the investigated treatments with AgNPs induced a significant change in proline content compared to the control. Among the treatments with AgNO_3_, only exposure to the 50 µM concentration resulted in a significant decrease in comparison to the control, which was successfully alleviated with cysteine addition. A comparison of treatments of each type of Ag showed that exposure to AgNP–CTAB had a higher impact than AgNO_3_ at the 50 µM concentration (Table 2).

Among the tested AgNP–PVP concentrations, neither induced a significant change in reduced glutathione (GSH) content in comparison to the control, while the addition of cysteine enhanced GSH production in the combined treatment with 50 µM AgNP–PVP compared to the control and AgNP–PVP alone. Exposure to the highest AgNP–CTAB concentration resulted in a reduced value when compared to the control and other AgNP–CTAB-treatments, but the addition of cysteine alleviated this effect. Two higher AgNO_3_ concentrations decreased GSH content in comparison to the control, although values were elevated in combined treatments with cysteine. If the effects of the corresponding concentrations of each type of Ag treatment are compared, a significantly higher impact on GSH content was observed at the highest (100 µM) concentration in AgNP–PVP compared to AgNP–CTAB (Table 2).

#### 2.4.2. Leaf

All investigated AgNP–PVP concentrations significantly increased the proline content to the similar value, which was successfully reduced by cysteine addition in the combined treatments. Exposure to all AgNP–CTAB treatments resulted in elevated values compared to the control, although it was statistically significant only for the 100 µM concentration. Cysteine addition significantly reduced the obtained value to the control level. Exposure to higher AgNO_3_ concentrations resulted in an increased proline content, although significantly only for the 100 µM concentration, which was successfully alleviated in the combined treatment with cysteine. A comparison between each type of Ag treatment revealed that the AgNP–PVP exhibited significantly stronger impact at the 25 µM concentration compared to AgNP–CTAB and AgNO_3_, while at the 50 µM concentration, AgNP–PVP had a stronger effect compared to AgNP–CTAB (Table 3).

Exposure to all AgNP–PVP treatments increased the GSH content compared to the control, although it was significant only for two lower concentrations (25 and 50 µM) and successfully alleviated upon combined exposure with cysteine. AgNP–CTAB treatments significantly increased the GSH content compared to the control in a dose-dependent manner. The addition of cysteine reduced the obtained values in all combined treatments, although only at the 50 and 100 µM concentrations was this decrease statistically significant compared to corresponding treatments without cysteine. Interestingly, the values obtained in all combined treatments were still significantly higher in comparison to the control. Exposure to AgNO_3_ also resulted in significantly elevated values compared to the control, although the lowest tested concentration (25 µM) resulted in the highest GSH content. Cysteine addition decreased the obtained values in all combined treatments, although statistically significant only at 50 and 100 µM concentrations in comparison to corresponding treatments without cysteine. If treatments with each type of Ag are compared, a significantly higher impact of AgNO_3_ compared to AgNP–PVP was observed at the lowest 25 µM concentration as well as at the highest 100 µM concentration, at which the highest effect was recorded for AgNP–CTAB (Table 3).

### 2.5. Antioxidant Enzyme Activity

#### 2.5.1. Root

**Figure 1:**
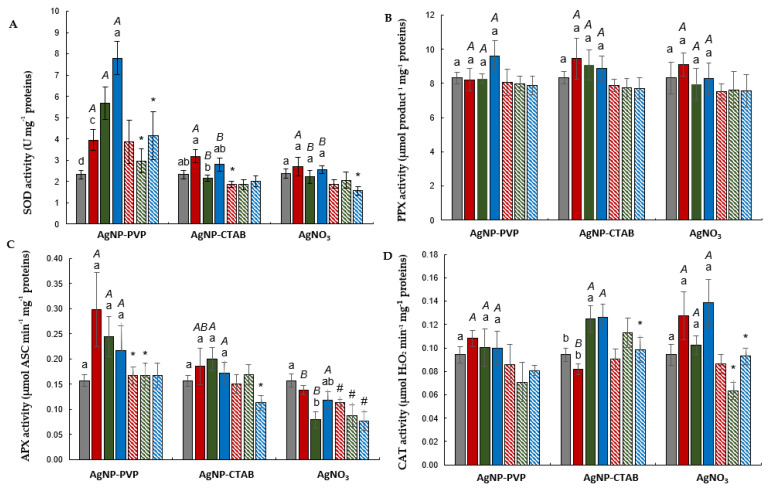
Activites of antioxidative enzymes (**A**) superoxide dismutase (SOD), (**B**) pyrogallol peroxidase (PPX), (**C**) ascorbate peroxidase (APX), and (**D**) catalase (CAT) in roots of tobacco plants 7 days after exposure to 25, 50, and 100 μM AgNP–PVP, AgNP–CTAB, and AgNO_3_, applied either alone or in combination with 125, 250, and 500 μM cysteine (cys). Values are the means ± SE of three biological replicates, each with six technical replicas. If columns are marked with different letters, the treatments are significantly different at *p* ≤ 0.05 (a two-way ANOVA followed by Duncan’s post hoc test); small letters mark the differences among different concentrations of the same treatment type as well as control; capital letters mark the differences among different treatment types of the same concentration; asterisk (*) denotes significant differences among treatments with and without cysteine of the corresponding concentration, while hash sign (#) denotes significant difference between each treatment with cysteine and control.

All investigated AgNP–PVP concentrations significantly elevated superoxide dismutase (SOD) activity in a dose-dependent manner. The addition of cysteine induced a decrease in the obtained values in all combined treatments, but significantly only at two higher concentrations. On the contrary, no significant difference in comparison to the control was recorded upon exposure to treatments with AgNP–CTAB or AgNO_3_. Comparing the effects of each type of Ag treatment, a higher impact on SOD activity was observed for AgNP–PVP in comparison to AgNP–CTAB and AgNO_3_ at higher concentrations (50 and 100 µM) (Figure 1).

The highest AgNP–PVP concentration (100 µM) induced an increase in pyrogallol peroxidase (PPX) activity, although not significantly; this was successfully diminished to the control value with cysteine addition. All AgNP–CTAB treatments elevated PPX activity in comparison to the control, although the increase was not statistically significant; the values were reduced to the control ones in combined exposure with cysteine. Among treatments with AgNO_3_, only the lowest concentration induced an increase compared to control, but again, not statistically different (Figure 1).

All treatments with AgNPs somewhat elevated APX enzyme activity, even though the increase was not significant in comparison to the control. However, increased values were alleviated with the addition of cysteine, which was significant upon exposure to all AgNP–PVP concentrations and the highest AgNP–CTAB one compared to treatments without cysteine of the corresponding treatment. Among AgNO_3_ treatments, the 50 µM concentration resulted in significantly decreased values. Combined treatments with AgNO_3_ and cysteine significantly reduced APX activity compared to the control. If treatments with each type of Ag treatment are compared, a higher impact on ascorbate peroxidase (APX) activity was observed for both types of AgNPs, particularly AgNP–PVP, in comparison to AgNO_3_ at lower tested concentrations (25 and 50 µM) (Figure 1).

None of the investigated treatments with AgNP–PVP exhibited a significant increase in catalase (CAT) activity in comparison to the control. On the contrary, exposure to higher concentrations (50 and 100 µM) of AgNP–CTAB resulted in significantly elevated values, which were alleviated with the addition of cysteine, although the significant decrease was recorded only upon the treatment with the highest concentration of the combined treatment compared to AgNP–CTAB alone. Values obtained in combined treatments were not significantly different in comparison to the control. As for the exposure to AgNO_3_, all investigated concentrations elevated CAT activity, although the increase was not significant when compared to the control. Cysteine addition resulted in lowered values, which was significant upon exposure to the combined treatments with 50 and 100 µM AgNO_3_ in comparison to corresponding treatments without cysteine. An analysis of the treatments with each type of Ag treatment showed a significant increase at the 25 µM concentration for AgNO_3_ in comparison to AgNP–CTAB (Figure 1).

#### 2.5.2. Leaf

**Figure 2:**
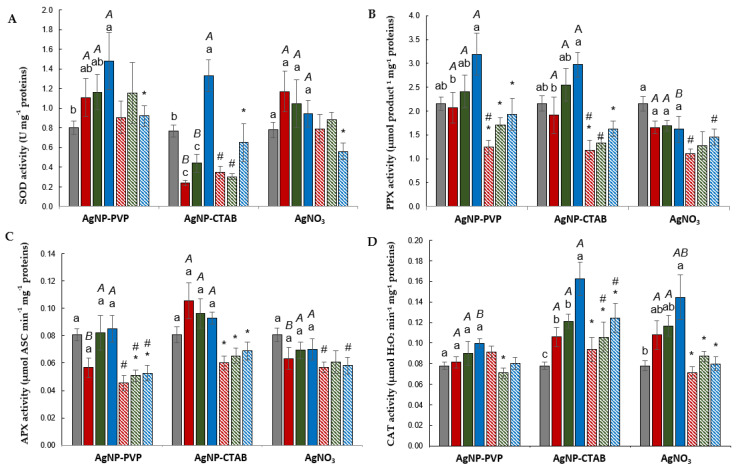
Differences in activites of (**A**) superoxide dismutase (SOD), (**B**) pyrogallol peroxidase (PPX), (**C**) ascorbate peroxidase (APX), and (**D**) catalase (CAT) in leaves of tobacco plants 7 days after exposure to 25, 50, and 100 μM AgNP–PVP, AgNP–CTAB, and AgNO_3_, applied either alone or in combination with 125, 250, and 500 μM cysteine (cys). Values are the means ± SE of three biological replicates, each with six technical replicas. If columns are marked with different letters, the treatments are significantly different at *p* ≤ 0.05 (a two-way ANOVA followed by Duncan’s post hoc test); small letters mark the differences among different concentrations of the same treatment type as well as control; capital letters mark the differences among different treatment types of the same concentration; asterisk (*) denotes significant differences among treatments with and without cysteine of the corresponding concentration, while hash sign (#) denotes significant difference between each treatment with cysteine and control.

All applied AgNP–PVP concentrations increased SOD activity, although only the value obtained upon exposure to 100 µM AgNP–PVP was statistically significant compared the to control; the addition of cysteine resulted in significantly lower SOD activity, which was again significant only at the highest tested concentration. Treatments with AgNP–CTAB induced different concentration-dependent responses; two lower concentrations (25 and 50 µM) significantly decreased SOD activity compared to the control, while the highest (100 µM) concentration resulted in a significantly elevated value. The addition of cysteine had no effect on SOD activity in combined treatments with 25 and 50 µM AgNP–CTAB, in comparison to AgNP–CTAB alone, and values were significantly lower in comparison to the control. However, cysteine addition significantly decreased the value at the highest applied concentration when compared to AgNP–CTAB alone. As for the exposure to AgNO_3_, the majority of the tested treatments, either alone or in combination with cysteine, had no significant effect on SOD activity; the only exception was the combination of 100 µM AgNO_3_ with cysteine, in which a statistically significant lower value compared to AgNO_3_ alone was recorded. An analysis of the effects of the corresponding concentrations of each type of Ag treatment showed a significant difference at the 25 and 50 µM concentrations for AgNP–CTAB in comparison to AgNP–PVP and AgNO_3_ (Figure 2).

PPX activity was not significantly affected upon exposure to any of the examined AgNP–PVP and AgNP–CTAB treatments compared to the control, although the 100 µM concentration resulted in a substantially higher value in comparison to corresponding treatments with 25 µM AgNP–PVP or AgNP–CTAB. Cysteine addition lowered PPX activity in all combined AgNP treatments compared to the values obtained upon exposure to AgNP–PVP or AgNP–CTAB alone, and in the 25 and 50 µM concentrations even with the control values. Treatments with AgNO_3_ had no significant effect on PPX activity; all obtained values were similar to the control, while cysteine addition resulted in a certain decrease, which was significant only at the 25 µM concentration. An analysis of the effects of the corresponding concentrations of each type of Ag treatment showed that exposure to both types of AgNPs exhibited significantly higher values than AgNO_3_ when applied at the 100 µM concentration (Figure 2).

None of the applied concentrations of each type of Ag treatment had a significant effect on APX activity in comparison to the control, while cysteine addition induced a significant decrease in the obtained values in combined treatments with both types of AgNPs when compared to treatments with AgNPs alone; moreover, upon exposure to the combination of AgNP–PVP and cysteine at all the tested concentrations, the obtained values were significantly lower in comparison to the control. If the effects of the corresponding concentrations of each type of Ag treatment are compared, a significantly higher impact on APX activity was observed for AgNP–CTAB in comparison to AgNP–PVP and AgNO_3_ at the lowest tested concentration (25 µM) (Figure 2).

None of the tested concentrations of AgNP–PVP, applied either alone or in combinations with cysteine, had a significant impact on CAT activity in comparison to the control. On the contrary, all treatments with AgNP–CTAB alone resulted in significantly elevated values, which was particularly pronounced at the highest concentration (100 µM). Cysteine addition managed to induce a decrease in CAT activity in combined treatments, although this decrease was significant only at the highest applied concentration compared to corresponding treatments with AgNP–CTAB alone; however, the obtained values were still significantly higher compared to the control. All AgNO_3_ treatments induced an increase in CAT activity, although only the 100 µM concentration resulted in a statistically significant increment compared to the control. The addition of cysteine successfully decreased CAT activity to values similar to the control in all combined treatments. If the effects of the corresponding concentrations of each type of Ag treatment are compared, a significantly higher impact on CAT activity was observed between AgNP–PVP (significantly lower) and AgNP–CTAB (significantly higher) at the highest tested concentration (Figure 2).

### 2.6. PCA Analysis

#### 2.6.1. Root

**Figure 3:**
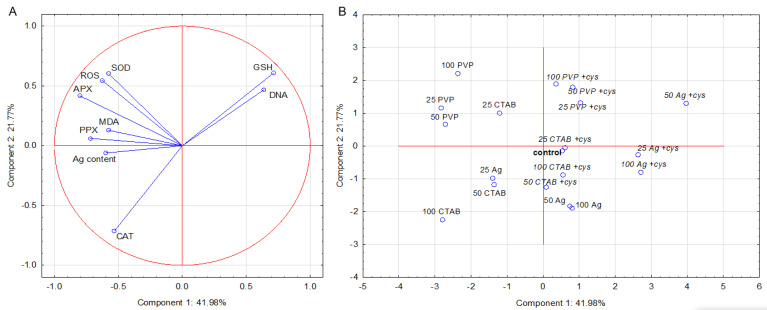
Principal component analysis of root data set obtained for tobacco plants 7 days after exposure to 25, 50, and 100 μM AgNP–PVP, AgNP–CTAB, and AgNO_3_, applied either alone or in combination with cysteine (+ cys). (**A**) loadings and (**B**) scores of the first two components (PCs). Silver (Ag) content, reactive oxygen species content (ROS), malondyaldehide content (MDA), glutathione content (GSH), DNA damage (DNA), activity of superoxide dismutase (SOD), ascorbate peroxidase (APX), pyrogallol peroxidase (PPX), and catalase (CAT) represent variables that contributed the most to the combined PC1/PC2.

In the case of the root data set, three principal components (PCs) were extracted, representing 93% of the variance. PC1 was mainly determined by APX and PPX activity with strong negative loadings and GSH with a positive loading, while PC2 was mostly determined by CAT activity with a negative loading and SOD, ROS, and GSH with positive loadings (Figure 3A). PC 3 was determined by MDA content with a high positive loading, but it did not contribute to the further separation of treatments. The corresponding score plot (Figure 3B) shows that PC1 contributed the most to the separation of treatments with AgNPs alone from the combined treatments with cysteine and the control, while PC2 contributed to the separation of AgNP–CTAB from AgNP–PVP, regardless of the presence of cysteine (Figure 3B). SOD activity and ROS content, variables with high positive loadings on PC2, were mainly responsible for a good separation of AgNP–PVP alone, while CAT activity with a high negative loading on PC2 mostly contributed to the separation of higher concentrations of AgNP–CTAB alone. GSH content and tail DNA, variables with high positive loadings on PC1, contributed to separation of combined treatment of AgNP–PVP with cysteine from other treatments.Interestingly, treatments with AgNO_3_ failed to separate well; higher concentrations were grouped together with combined treatments of AgNP–CTAB with cysteine and control.

#### 2.6.2. Leaf

**Figure 4:**
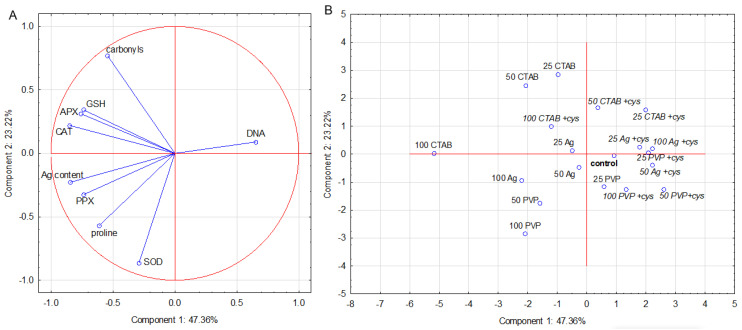
Principal component analysis of leaf data set obtained for tobacco plants 7 days after exposure to 25, 50, and 100 μM AgNP–PVP, AgNP–CTAB, and AgNO_3_, applied either alone or in combination with cysteine (+cys). (**A**) loadings and (**B**) scores of the first two components (PCs). Silver (Ag) content, reactive oxygen species content (ROS), malondyaldehide content (MDA), glutathione content (GSH), DNA damage (DNA), activity of superoxide dismutase (SOD), ascorbate peroxidase (APX), pyrogallol peroxidase (PPX), and catalase (CAT) represent variables that contributed the most to the combined PC1/PC2.

Results for leaf data set show that two PCs explained 70% of the variation. CAT and APX activities as well as GSH and Ag content were major contributors to PC1, while SOD activity and carbonyl content contributed the most to PC2 (Figure 4A). The corresponding score plot (Figure 4B) shows that PC1 separated most treatments with AgNP alone (with the exception of the 25 µM AgNP–PVP) and AgNO_3_ alone from the combined treatments with cysteine (with the exception of the 100 µM AgNP–CTAB) and the control. PC2 contributed to the partial separation of all AgNP–CTAB treatments from most AgNP–PVP ones, regardless of the presence of cysteine (Figure 4B). CAT and APX activities as well as Ag and GSH contents, variables with high positive loadings on PC1, and carbonyls with the highest positive loading on PC2, were mainly responsible for the separation of treatments with AgNP–CTAB alone from other treatments. On the other hand, SOD activity and proline content, with a high negative loading on PC2, mostly contributed to the separation of treatments with higher concentrations of AgNP–PVP and AgNO_3_ alone.

### 2.7. AgNP Localisation in Root Cells

After exposure to 100 µM concentration of either AgNP–PVP or AgNP–CTAB, AgNPs were visible as black dots mainly in the epidermal root cells (Figures S1A and S2A). Therefore, root cells were further examined by TEM-EDX. Figures S1 and S2 show that AgNPs were localized in cell cytoplasm and in the intermembrane space. The EDX scan confirmed that the particles found in the TEM images contained silver (Figures S1C and S2D), which proves the direct uptake of both AgNP–PVP and AgNP–CTAB and their accumulation in the root cells.

## 3. Discussion

### 3.1. Roots

Previous studies have proposed that AgNP phytotoxicity has been mainly attributed to the excess generation of ROS, leading to the induction of oxidative stress [2,20]. Indirectly, the dissolution of Ag^+^ ions from AgNPs as well as the intrinsic properties of their surface coatings can affect AgNP toxicity and can contribute to ROS overproduction in the promotion of oxidative stress [2].

In this study, oxidative stress parameters revealed that the lowest concentration of AgNP–PVP and AgNO_3_ significantly induced ROS formation when compared to the control tissue, while two higher AgNP–PVP and the lowest AgNP–CTAB concentrations resulted in elevated values although not statistically significant. In our previous study, enhanced ROS formation in root tissue was recorded after exposure of tobacco plants to AgNO_3_, while no changes were recorded after treatments with AgNP–citrate [15], which suggests that the coating might contribute to the intensity of ROS formation [14]. Furthermore, none of the applied concentrations of neither AgNP–PVP nor AgNP–CTAB induced a significant increase in MDA and protein carbonyl content, which is in good correlation with our previous findings obtained with AgNP–citrate [15]. On another hand, in the study on wheat (*Triticum aestivum*) roots, MDA content decreased upon treatment with uncoated AgNPs, but increased after exposure to AgNO_3_, compared to the control [40]. In the investigations from other authors, it was reported that the exposure of wheat cultivars [41], rice [42], and *Arabidopsis* [43] to AgNP–citrate increased lipid peroxidation and protein oxidation. Moreover, in *Allium cepa* roots, significantly higher MDA and protein carbonyl contents in correlation with high ROS content were recorded after treatment with citrate-, PVP- and CTAB-coated AgNPs applied in 50, 75, and 100 μM concentrations [14], which suggests that AgNP-induced phytotoxic effects are also dependent on the tested plant species beside the AgNP coating.

The current results reveal that neither AgNPs nor AgNO_3_ induced significant DNA damage in tobacco roots compared to the control, although the highest concentrations of AgNP–PVP and AgNO_3_ slightly increased the % of tail DNA. Ghosh et al. [44] reported a dose-dependent increase in the extent of DNA damage in *A. cepa* and *N. tabacum* roots after exposure to AgNP–PVP. However, when in a study of Cvjetko et al. [14] differently coated AgNPs were applied on *A. cepa* roots, no DNA damage was observed with AgNP–citrate, while AgNP–CTAB exhibited significantly higher DNA damage compared to AgNP–PVP, which has been at least partially ascribed to higher Ag uptake upon exposure to AgNP–CTAB in comparison to AgNP–PVP. These findings do not corroborate the results of the current study, in which significantly higher Ag accumulation was obtained in the majority of the AgNP–CTAB treatments compared to the AgNP–PVP ones, but the AgNP–CTAB treatment failed to show any impact on the integrity of the DNA molecule. In the roots of tobacco plants citrate-coated AgNP also failed to induce DNA damage evaluated by the Comet test [15], which indicates that in addition to surface coating applied for AgNP stabilization, AgNP-induced genotoxicity might also be dependent on the tested plant model [24].

Among the investigated antioxidant enzymes, SOD constitutes the first line of defense against ROS by neutralizing the superoxide radical (O_2_^−•^) to hydrogen peroxide (H_2_O_2_) and thus plays a central role in preventing damage to biologically important molecules [20]. In this study, SOD activity in roots was found to be enhanced upon exposure to both types of AgNPs, particularly to PVP-coated ones, which is in a good correlation with enhanced ROS formation found upon exposure to AgNP–PVP, since the dihydroethidium (DHE) test applied for ROS detection predominantly measures the formation of the O_2_^−•^ [14] Moreover, activities of PPX and CAT were also found to be elevated with both types of AgNP treatments, although this was more enhanced in AgNP–CTAB, which suggests that both types of AgNPs induce the activation of root cell antioxidant machinery. These results correlate well with the observed lack of oxidative damage to biomolecules after most AgNP treatments, but also indicate that activation of certain components is coating-dependent. In our previous study, citrate-coated AgNPs in general did not induce significant changes in activity of SOD and PPX in roots of tobacco plants, while lower concentrations induced higher CAT activity [15]. Moreover, when the effects of differently coated AgNPs on *A. cepa* roots were investigated, it was found that unlike AgNP–citrate, both AgNP–PVP and AgNP–CTAB elevated PPX activity, which is in accordance with the results obtained in the current study. Interestingly, APX activity was not found to be significantly affected in tobacco root cells by either AgNP–PVP or AgNP–CTAB, while exposure to citrate-, PVP- and CTAB-coated AgNPs mostly decreased activity of this enzyme in *A. cepa* roots, which again suggests that AgNP-provoked effects are also dependent on the plant species. As for the effects of AgNO_3_ exposure in the current study, the only increase in antioxidant enzyme activity was found for CAT compared to the control, although the values didn’t exceed those obtained upon exposure to AgNP–CTAB of the corresponding concentration. This is an interesting result, since in our previous studies, AgNO_3_ induced more prominent effects in antioxidant enzyme activities compared to AgNP–citrate in tobacco roots [15]. This discrepancy in the obtained results can be ascribed to the different exposure media, which was ultrapure water in our previous study [15], compared to ½ strength nutrient medium applied in the current one. Specifically, Ag^+^ ions dissolved from AgNO_3_ can be bound by components of the nutrient medium to form Ag salts [2], thus reducing the uptake of potentially toxic Ag^+^ ions, as indicated by the results of the Ag accumulation in roots cells, where significantly lower values were recorded for AgNO_3_ treatments compared to the corresponding concentrations of AgNP–PVP and AgNP–CTAB.

In addition, enzymatic mechanism, plants counteract oxidative stress by using small nonenzymatic antioxidant molecules, such as proline and GSH, in the process of ROS detoxification [2,45,46]. In this study, the levels of reduced GSH were mostly dependent on the concentration of the treatment and the type of Ag source; namely, AgNP–PVP showed no significant impact, whereas two higher AgNO_3_ concentrations and only the highest AgNP–CTAB concentration significantly decreased the GSH levels compared to the control. A similar trend was observed in the roots of *Camelina sativa* seedlings treated with uncoated AgNPs, where the GSH content increased at lower concentrations of AgNPs and reduced at higher concentrations of AgNPs [47]. One of the possible explanations for the decrease in the GSH levels upon AgNP treatment is that silver could inhibit the activity of glutathione reductase (GR), which is responsible for maintaining the supply of GSH, and induce glutathione S-transferase (GST) activity, which catalyzes the conjugation of the GSH thiol group with other electrophilic compounds [48], making it unavailable for the reaction with Ellman’s reagent [49]. Such a decrease in the GR activity and an increase in GST activity was reported in *Hordeum vulgare* roots treated with AgNPs [50], while a decrease in GR activity and GSH content was observed in *Solanum tuberosum* roots exposed to AgNP–PVP [51]. One other explanation is the ability of silver to bind to the nucleophilic SH group of GSH [47]. Furthermore, the content of proline in tobacco roots did not significantly change in response to AgNP treatments and only declined significantly upon exposure to 50 µM AgNO_3_. In the study on *Medicago sativa* roots exposed to AgNPs, only the highest applied concentration, which was more than 18× higher than the highest concentration used in our study, induced a significant decrease in the proline content, whereas treatments of lower concentrations induced an increase in proline content compared to the control, which was, however, statistically insignificant [52]. The decline in the proline content could be due to the activation of the proline degradation pathway, in which the oxidation of one molecule of L-proline yields approximately 30 ATP equivalents, thereby providing important energy for the cell to cope with the stress conditions [53]. One other possibility is the complexation of proline with Ag^+^ ions, forming a zwitterionic proline-Ag complex [54], which could sterically interfere with the reaction of the imino group of proline with the ninhydrin reagent, used for proline determination [55].

To our knowledge, the investigation of the cysteine ability to alleviate the toxic effects of AgNPs in terms of cellular response to oxidative stress in plants or in green algae has received little to almost no attention to date. By binding Ag^+^ ions dissociated from AgNPs, cysteine could reduce the toxicity resulting from Ag^+^ ions [20]. In this work, the addition of cysteine mostly alleviated AgNP- and AgNO_3_-induced effects, suggesting that their impact is at least partly a consequence of the dissociation of Ag^+^ ions from nanoparticles. This is also indicated by the PCA analysis, which showed the separation of treatments with AgNP alone from combined treatments with cysteine and the control. However, the Ag content in the root tissue shows a higher uptake after the treatment with AgNPs compared to AgNO_3_, and TEM-EDX confirmed AgNP presence in the root cells. Moreover, the Ag content in roots remained high, even after the addition of cysteine, without causing any prominent effects. A PCA analysis showed that treatments with AgNP–PVP were largely separated from those with AgNP–CTAB regardless of cysteine. Taking all results together, it seems that 7-day exposure of adult tobacco plants to AgNPs causes low toxicity to root tissue and that some of the measured plant responses are dependent on the AgNP coating.

### 3.2. Leaves

Oxidative stress parameters measured in leaf tissue revealed the slightly toxic effects of AgNPs, which could be related to the Ag accumulation in leaves, although it was many times lower than in roots after both AgNP and AgNO_3_ treatments. This result indicates that the majority of the accumulated Ag remained in the root cells and that only a small portion of Ag was translocated to the leaves. These findings are in a good correlation with results from our previous studies in which higher Ag uptake was recorded in roots compared to leaves upon exposure of tobacco plants to AgNP–citrate and AgNO_3_ in ultrapure water [15]. Moreover, no AgNPs were detected in the leaf cells of tobacco plants treated neither with AgNP–citrate in ultrapure water [15] nor with citrate-, PVP- or CTAB-coated AgNP in a liquid 1/2 strength nutrient medium [25], which was used in the current study.

In leaves, none of the applied AgNP concentrations of either AgNP–PVP or AgNP–CTAB induced an increase in ROS formation and DNA damage, which is in accordance with our previous study performed with citrate-coated AgNPs [15]. On the contrary, Gosh et al. [44] reported DNA damage in tobacco leaves after exposure to 25- 75 mg L^−1^ AgNP–PVP, although the effects were not as prominent as in the roots. Furthermore, Lovecka et al. [56] found an increased average median tail moments by employment of the Comet assay in leaves of tobacco plants exposed to AgNP–citrate higher than 30 mg L^−1^, which is almost 3× higher than the highest concentration tested in this study.

Contrary to the results obtained previously with AgNP–citrate [15], in this study, MDA and particularly protein carbonyl content increased upon exposure to AgNP–CTAB, which was not recorded in the treatments with either AgNP–PVP or AgNO_3_. Similarly, treatment with AgNP–PVP did not induce changes in the MDA content in *Spirodela polyrhiza*. These results once again indicate very prominent phytotoxic effects of CTAB-coated AgNPs. Higher toxic effects of CTAB-coated AgNPs on photosynthesis have already been reported by Peharec Štefanić et al. [25], who correlated them with their surface charge, since stability analyses showed that AgNP–CTAB kept their positive charge in a liquid 1/2 strength nutrient medium, while the ζ potential became even more negative with time for AgNP–PVP. Cvjetko et al. [14] have also reported that positively charged AgNP–CTAB had a more severe impact on oxidative stress appearance in onion root cells than negatively charged citrate or nonionic PVP coatings, probably due to attachment of positively charged AgNPs to the negatively charged plant cell walls. A significantly higher toxicity of positively charged AgNP–cystamine compared to AgNP–citrate was also reported for two wheat varieties [57]. As for the treatments with AgNO_3_, our results reveal they had the weakest impact on the investigated oxidative stress parameters in leaf cells, which is in accordance with findings from our previous study in which AgNO_3_ exposure had the least impact on tobacco leaf (ultra)structure, photosynthesis, and pigment content compared to PVP- and CTAB-coated AgNPs [25].

Among the investigated antioxidant enzymes, all of them were found to be sensitive to applied AgNP treatments, while only CAT responded to AgNO_3_. At the 100 µM concentration, both types of AgNPs induced significant SOD and PPX activity elevation. The increase in SOD and PPX activities was also found in frond cells of *S. polyrhiza* upon exposure to AgNPs coated with GA and PVP, while CAT activity was significantly elevated only after the treatments with AgNP–GA [45]. Similar results were obtained upon exposure of castor bean seedlings to AgNP–PVP, which elevated SOD and PPX activity at all applied AgNP concentrations. Interestingly, our results show that two lower AgNP–CTAB concentrations resulted in a significant reduction of SOD activity. Downregulation of Fe–SOD was reported in tobacco leaves upon plant exposure to AgNP–citrate [58]. In the study of Cvjetko et al. [15], citrate-coated AgNPs had no significant impact on SOD, PPX, and APX activity in the leaves of tobacco plants, while all of the applied AgNP–citrate concentrations significantly decreased CAT activity [15]. On the contrary, in this study, all AgNP–CTAB and the highest AgNO_3_ concentration significantly induced CAT activity, which suggests that the response of the plant antioxidant system to AgNP-induced stress might be coating-dependent. Lower concentrations of AgNO_3_ had no significant impact in comparison to the control, which is in positive correlation with the results obtained in Cvjetko et al. [15].

As for the nonenzymatic antioxidant defense system, exposure to all AgNP–PVP concentrations activated proline synthesis. Interestingly, among the AgNP–CTAB and AgNO_3_ concentrations, only the highest one resulted in significantly elevated values compared to the control. Previous studies reported significantly increased foliar proline amount in wheat [59] and rice [42] upon exposure to citrate- and PVP-coated AgNPs. An increase in proline content is considered to be a systematic response to metal toxicity since proline is an important osmolyte, which acts as a metal chelator and thus may detoxify ROS overproduction induced by both AgNPs and AgNO_3_ [2,60]. Total glutathione was found to be an even more responsive parameter, since the values were significantly higher upon all tested treatments, although it was more pronounced after exposure to AgNP–CTAB and AgNO_3_ compared to AgNP–PVP. GSH acts as a scavenger of ROS [61] and, as a substrate in the synthesis of phytochelatins, can be indirectly involved in heavy metal detoxification [2]. A concentration-dependent increase in GSH content upon exposure to AgNP–PVP was reported in duckweed *S. polyrhiza*, where it efficiently alleviated oxidative stress, probably by chelating Ag^+^ ions released from AgNPs [45]. In general, treatments with AgNPs had a prominent effect on the activation of both enzymatic and nonenzymatic components of antioxidant machinery in leaves, which could be correlated with the deterioration of photosynthetic activity found previously [25], thus implying the need for enhanced protection against ROS due to overexcitation of photosystems induced by AgNP exposure.

Most effects induced by both AgNPs as well as AgNO_3_ were alleviated, at least partially, with the addition of cysteine. Previous studies showed that the addition of cysteine improved early growth parameters of tobacco seedlings in comparison with the AgNP–CTAB alone [34], indicating a fast removal of surface coating and showing that CTAB is a relatively labile ligand [2]. Furthermore, the addition of cysteine completely prevented the negative effects on photosynthetic yield after exposure to nine differently coated AgNPs in green algae *Chlamydomonas reinhardtii* [62]. As in the roots, the results of the PCA analysis showed that most treatments with AgNP and AgNO_3_ alone separated from the combined treatments with cysteine and the control, thus confirming the alleviating effect of cysteine. However, treatments with AgNO_3_ had a weaker impact on adult plants than AgNPs. Moreover, separation of treatments with AgNP–CTAB alone from other treatments as well as separation of treatments with higher concentrations of AgNP–PVP and AgNO_3_ alone revealed by PCA analysis suggests distinct responses to differently coated AgNP.

Comparing the response of the roots and the shoots, all tested treatments induced a more prominent effect and stronger antioxidant response in leaves, in spite of a much higher Ag accumulation in roots. Moreover, the activation of the specific components of the antioxidant system after treatments was organ-specific; i.e., an increase in nonenzymatic antioxidants was observed in leaves, whereas the activity of peroxidase enzymes (APX and PPX) was elevated in roots. The results suggest that different plant organs engage specific responses to cope with the Ag-induced oxidative stress.

## 4. Materials and Methods

### 4.1. AgNP Synthesis and Characterization

All chemicals were purchased from Sigma-Aldrich, St. Louis, MO, USA, unless stated differently and were at least of analytical purity. For all synthesis procedures, ultrapure water (ion-free Milli-Q water, 18.2 MΩ·cm, Merck Millipore, Billerica, MA, USA) was used.

Syntheses of AgNPs coated with polyvinylpyrrolidone (AgNP–PVP) and cetyltrimethylammonium bromide (AgNP–CTAB) were performed as reported in Peharec Štefanić et al. [25]. Both AgNP solutions were stored at 4 °C until analysis. Physical and chemical characteristics of AgNP–PVP and AgNP–CTAB stock solutions were principally analyzed as previously reported [25]. Formation of AgNPs in both solutions was confirmed by the presence of a SPR peak using a UV-Vis spectrophotometer (Unicam, Cheshire, UK). The size (hydrodynamic diameter) and charge (ζ potential) of AgNPs were measured using Zetasizer Nano ZS (Malvern, UK) equipped with green laser (532 nm). Intensity of scattered light was detected at the angle of 173°. All measurements were conducted at 25 °C. For data processing, Zetasizer software version 6.32 (Malvern Panalytical, Malvern, UK) was used. The AgNP hydrodynamic diameters are given as the average value of 10 measurements (mean ± S.D., n = 10) and are reported as the volume size distributions, while AgNP ζ potentials are reported as the average of five measurements (mean ± S.D., n = 5). The concentration of Ag^+^ deriving from AgNP dissolution in ultrapure water was determined by centrifugal ultrafiltration (Millipore Amicon Ultra-4 3K, Merck Millipore, Billerica, MA, USA) through a 3 kDa molecular weight cutoff membrane. Total Ag concentrations in AgNP colloidal suspensions and filtrates were determined in acidified solutions (10% HNO_3_) using an ELAN DRC-e (Perkin Elmer, Waltham, MA, USA) inductively coupled plasma mass spectrometer (ICP-MS). To calculate Ag concentration, calibration curve obtained with a set of standards of known concentrations was used. Detection limit and limit of quantification (LOQ) were 0.2 and 1 mg kg^−1^, respectively. In addition, synthesized and purified AgNP–PVP and AgNP–CTAB were visualized using a monochromated TF20 (FEI TecnaiG2) as in our previous study [25].

### 4.2. Plant Material and Exposure Experiments

Tobacco (*Nicotiana tabacum* L. cv Burley) plants were cultivated in conditions of plant tissue culture as previously described [15,25], with minor modifications. Seeds were firstly surface-sterilized with 50% (*v*/*v*) sodium hypochlorite (Kemika, Zagreb, Croatia) for 15 min, washed 3× with deionized H_2_O, and subsequently germinated on liquid Murashige and Skoog (MS) [63] nutrient medium supplemented with 500 mg L^−1^ MES (2-(N-morpholino)ethanesulfonic acid) and 1.5 g L^−1^ sucrose. Sterilized tobacco seeds were transferred to an Erlenmeyer flask (150 mL) containing 5 mL of autoclaved liquid 1/2 strength MS medium, and were germinated on a laboratory shaker in the growth chamber (16/8 light/dark cycle, light intensity 90 µmol m^−2^ s^−1^, and temperature 24 °C) for 3 weeks. Tobacco seedlings were then transferred into a bigger glass container containing 50 mL of liquid 1/2 strength MS medium and were supported by glass holders to ensure that only roots were immersed into the liquid medium. Seedlings were left to grow in the growth chamber under aseptic conditions in the aforementioned conditions until adult plants with a well-developed root system and shoots with differentiated leaves were obtained [25].

For exposure experiments, adult plants were transferred to a liquid 1/2 strength MS medium supplemented with AgNP–PVP, AgNP–CTAB, or AgNO_3_, alone and in combination with cysteine (applied in a cysteine:silver molar ratio of 5:1), to obtain 25, 50, and 100 µM concentrations of silver and 125, 250, and 500 µM concentrations of cysteine, respectively. Control plants were cultured in a liquid 1/2 strength MS medium without silver. Control and treated plants were grown for 7 days in the growth chamber under the aforementioned conditions. The experiment was performed three times with six replicates for each treatment. Prior to subsequent measurements, roots and leaves were detached and analyzed separately.

### 4.3. Ag Content Measurements

Measurement of Ag content in treated plant material was performed as previously reported [25]. Briefly, separated roots and leaves of exposed as well as control tobacco plants were dried in a microwave oven for 24 h at 80 °C and subsequently powdered using a mortar and pestle. Tissue was digested in a microwave oven (ETHOS SEL Milestone, Shelton, CT, USA) by applying the EPA 3051a method, first in 10 mL of concentrated nitric acid (HNO_3_) at 130 °C for 10 min and then at 180 °C for another 15 min. The second step was digestion in 1 mL of H_2_O_2_ at 85 °C for 5 min and then at 130 °C for 4 min. The samples were cooled and subsequently diluted with 1% (*v*/*v*) HNO_3_ up to a final volume of 50 mL for determination of the total Ag content, ELAN DRC-e ICP-MS instrument was applied. For calculation of the Ag concentration, a calibration curve obtained with a set of standards of known concentrations was used. The detection limit and limit of quantification (LOQ) were 0.05 and 0.1 mg kg^−1^, respectively. Spike recovery tests were 95.6, 95.2, and 96.5% for roots of AgNP–PVP-, AgNP–CTAB- and AgNO_3_-treated plants, respectively, and 96.2%, 96.6%, and 96.8% for leaves of AgNP–PVP-, AgNP–CTAB- and AgNO_3_-treated plants, respectively. Ag content was expressed as micrograms of Ag per gram of tissue dry weight.

### 4.4. Protein Extraction

Prior to protein extraction, fresh roots and leaves were frozen at −80 °C and subsequently lyophilized. Lyophilized tissue (500 mg of roots and 350 mg of leaves) was ground by mortar and pestle in 1.5 mL of 100 mM potassium phosphate buffer, pH 7.0. The 50 mg of insoluble PVP was added to the plant material prior to grinding. Obtained homogenates were centrifuged for 15 min at 20,000× *g* at 4 °C, after which supernatants were collected and recentrifuged for 45 min at 20,000× *g* at 4 °C. Protein concentration was measured according to the Bradford method [64] using a bovine serum albumin (BSA) as a standard. These supernatants were subsequently used for ROS determination, carbonyl quantification, and assays of enzymatic activity.

### 4.5. ROS Determination

Level of ROS in root and leaf cells was determined using a fluorescent probe DHE [14,65]. A total of 50 μL of protein extract of each sample (Section 4.4) was mixed with 50 μL 20 μM DHE in a microtiter plate. Fluorescence intensity was measured immediately without incubation from the same plate using an Infinite 200 PRO microplate reader (Tecan, Zürich, Switzerland) at an excitation wavelength of 520 nm and an emission wavelength of 600 nm. Results are presented as relative intensity compared to relevant control (nontreated sample).

### 4.6. Malondialdehyde and Protein Carbonyl Content

The level of lipid peroxidation was determined by measuring the MDA content according to the modified method of Heath and Packer (1968) [66]. A total of 200 mg of fresh roots and 200 mg of fresh leaves were homogenized in 1.3 mL of 0.3% (*w*/*v*) 2-thiobarbituric acid (TBA) prepared in 10% (*w*/*v*) trichloroacetic acid (TCA) and incubated for 30 min at 95 °C. Mixtures were cooled in an ice bath and subsequently centrifuged for 1 h at 20,000× *g* and 4 °C. The absorbance of the supernatant was measured at 532 nm, while subtraction of the absorbance recorded 600 nm was performed for correction of nonspecific turbidity. Lipid peroxide content was calculated by applying the MDA molar absorption coefficient (155 mM^−1^ cm^−1^) and expressed as nanomole per gram of fresh weight.

Protein carbonyl quantification was determined according the method of Levine et al. [67], using the reaction with 2,4-dinitrophenylhydrazine (DNPH). A total of 20 μL of protein extract of each sample (Section 4.4) was diluted in 180 µL of 100 mM potassium phosphate buffer pH 7.0, combined with 300 μL of 10 mM DNPH in 2 M HCl or with 300 μL of 2 M HCl, so that each sample had its reference, and incubated for 1 h at room temperature protected from light. Subsequently, proteins were precipitated with 500 μL of cold 10% (*w*/*v*) TCA, after which samples were cooled at −20 °C and centrifuged for 10 min at 20,000× *g* and 4 °C. Obtained pellets were washed with 500 μL of ethanol/ethylacetate (1/1 *v*/*v*) mixture 3× in order to remove the excess reagent. Precipitated proteins were dissolved in 6 M urea in 20 mM potassium phosphate buffer (pH 2.4) in an ultrasonic bath. Absorbance was measured at 370 nm. For protein recovery estimation, the absorbance of each sample was measured at 280 nm. Protein carbonyl content was calculated using a molar absorption coefficient for aliphatic hydrazones of 22 mM^−1^ cm^−1^ and expressed as micromole per milligram of proteins.

### 4.7. Comet Assay

The Comet assay was performed according to Gichner et al. [68] with previously published modifications [14,69]. Briefly, nuclei form root and leaf cells were mechanically isolated in 400 mM Tris-HCl (pH 7.5) at 4 °C and mixed in equal volumes (50 μL) with low melting point agarose (LMP, 1% (*w*/*v*)). After 10 min of denaturation (for DNA unwinding) and 20 min of electrophoresis (0.8 V cm^−1^ and 300 mA) in a freshly prepared buffer (1 mM Na_2_EDTA and 300 mM NaOH, pH 13), slides were neutralized, air-dried, and subsequently stained with 70 mL ethidium bromide (20 mg mL^−1^) for 5 min. For measurement of the tail DNA percentage (% tDNA), as the primary measure of DNA damage, the computerized image analysis system Komet version 5 (Kinetic Imaging Ltd., Liverpool, UK) was applied.

### 4.8. Activity Assays of Antioxidant Enzymes

All enzymatic assays were performed at 25 °C, and all spectrophotometric analyses were conducted in a UV/visible spectrometer (ATI UNICAM UV4, Cambridge, UK).

SOD (E.C. 1.15.1.1) activity was determined according to the method published by Beauchamp and Fridovich [70]. The reaction mixture consisted of 13 mM methionine, 75 μM nitroblue tetrazolium (NBT), 0.1 M ethylenediaminetetraacetic acid (EDTA), and 2 mM riboflavin, to which different volumes of protein extracts (Section 4.4) in 50 mM phosphate buffer (pH 7.8) were added. Mixtures were kept for 8 min in a transilluminator (15 W), after which the formazan formation produced by NBT photoreduction was read at 560 nm. One unit of SOD activity was defined as the amount of enzyme required to generate 50% inhibition of the NBT reduction rate. Activity was expressed as units of SOD activity per milligram of protein.

PPX (E.C. 1.11.1.7) activity was estimated by measuring the absorbance increase at 430 nm as a result of the pyrogallol oxidation (ɛ = 2.6 mM^−1^ cm^−1^) [71]. The reaction mixture contained 50 mM potassium phosphate buffer (pH 7.0), 20 mM pyrogallol and 1 mM H_2_O_2_, supplemented with 20 μL of protein extract (Section 4.4). PPX activity was calculated as micromole of purpurogallin (product of pyrogallol oxidation) per minute per milligram of protein.

APX (E.C. 1.11.1.11) activity, was evaluated by the decrease in absorbance measured at 290 nm (ɛ = 2.8 mM^−1^ cm^−1^) [71]. The reaction mixture consisted of 50 mM potassium phosphate buffer (pH 7.0), 0.1 mM ascorbate, and 0.12 mM H_2_O_2_, supplemented with 180 μL of protein extract (Section 4.4). APX activity was expressed as micromole of ascorbate oxidized per minute per milligram of protein.

CAT (E.C. 1.11.1.6) activity, the decrease in absorbance at 240 nm (ɛ = 36 mM^−1^ cm^−1^) was measured every 10 s during 2 min, as described by Aebi [72]. The reaction mixture was composed of 50 mM potassium phosphate buffer (pH 7.0) and 10 mM H_2_O_2_, to which 30 μL of protein extract (Section 4.4) was added. CAT activity was calculated as micromole of decomposed H_2_O_2_ per minute per milligram of protein.

### 4.9. Proline and Glutathione Contents

Proline content was determined using the ninhydrin reaction method [55]. Briefly, approximately 150 mg lyophilized tissue samples were homogenized in 1.5 mL of 3% (*w*/*v*) sulfosalicylic acid, and the extract was centrifuged for 10 min at 20,000× *g*. After mixing sample with acid ninhydrin and glacial acetic acid in a 1:1:1 ratio, the resulting mixture was heated at 95 °C for 1 h, and the reaction was stopped in an ice bath. The mixture was extracted with 1.5 mL of toluene, and the absorbance was measured at 520 nm. Proline concentration was determined using a calibration curve and expressed as micromoles proline per gram dry weight.

GSH content was determined according to the method of Salbitani et al. [73] with minor adjustments. Lyophilized plant material (150 mg) was ground with mortar and pestle in 1.5 mL of 3% (*w*/*v*) salicylic acid (SA). The 50 mg of PVP was added to the plant material prior to grinding. Extracts were centrifuged for 10 min at 20,000× *g* at 4 °C, after which supernatants were collected and kept on ice until analyses. For GSH measurement, 100 μL of samples was mixed with 750 μL of reaction mixture (1.5 mg mL^−1^ 5,5 -dithio-bis-(2-nitrobenzoic acid); 0.1 M potassium phosphate buffer, pH 7.0; and 1 mM EDTA) and left to incubate at 25 °C for 20 min. Subsequently, absorbance was measured at 412 nm, and the GSH concentration was read using the calibration curve obtained with a set of GSH standards (0.785–25 μM) of known concentrations.

### 4.10. Microscopy Analyses

Analyses of AgNP localization in roots were performed in plants exposed to 100 µM AgNP–PVP and 100 µM AgNP–CTAB. Small pieces of root tissue were fixed with 1% (*w*/*v*) glutaraldehyde in 50 mM cacodylate buffer (pH 7.2) for 1 h at +4 °C. After that, they were washed with cold 50 mM cacodylate buffer (pH 7.2) twice and postfixed with 1% (*w*/*v*) osmium tetroxide in the same buffer for 1 h at +4 °C, after which a 10 min wash in ice-cold water followed. After dehydration in a graded series of ethanol, the tissue was embedded in Spurr’s resin. Ultrathin sections were examined using monochromated TF20 (FEI Tecnai G2, FEI, Hillsboro, OR, USA) TEM.

### 4.11. Statistical Analysis

The data were analyzed using the STATISTICA 14.0 (TIBCO Software Inc., Palo Alto, CA, USA) software package. All errors are indicated as standard errors (SE). Variations between treatments for the respective plant responses were tested using a one-way ANOVA analysis of variance, followed by post hoc Duncan’s multiple range test and critical ranges. Differences between means were considered statistically significant at *p* < 0.05. Principal component analysis (PCA) was performed to evaluate the most important responses in roots and leaves of tobacco plants exposed to different concentrations of AgNP–PVP, AgNP–CTAB, and AgNO_3_, either alone or in combination with cysteine, as well as to discriminate the responses to individual Ag treatments. PCA was applied to the standardized data sets, and the principal components (PCs) and contributions of each trait to PCs were determined. Responses that made the biggest contributions to the combined PC1/PC2 were used for grouping.

## 5. Conclusions

Ag uptake in both roots and leaves of tobacco plants was found to be higher upon exposure to both types of AgNPs compared to AgNO_3_, which can be correlated with a somewhat stronger impact on oxidative stress parameters caused by silver in a nanoparticle form. However, no severe damage to important biomolecules was observed after either type of treatment, although mobilization of antioxidant machinery was recorded, which was more pronounced upon exposure to AgNPs compared to AgNO_3_. These findings suggests that both types of AgNPs did induce oxidative stress, which was, however, successfully eliminated by employment of enzymatic as well as nonenzymatic antioxidants, thus preventing the impairment of lipids, proteins, and nucleic acids. Interestingly, a stronger activation of both enzymatic and nonenzymatic components of antioxidant machinery was recorded in leaves compared to roots upon exposure to both AgNP–PVP and AgNP–CTAB, although surface coatings modulated the activation of the specific components. SOD activity and proline content were more enhanced in treatments with AgNP–PVP, while the enzymes CAT and APX as well as the nonenzymatic antioxidant GSH were more activated upon exposure to AgNP–CTAB. Most effects induced by AgNPs or AgNO_3_ were alleviated with the addition of cysteine, which indicates that the impact of the nanoparticulate form of silver can at least be partially ascribed to dissociated Ag^+^ ions.

## Figures and Tables

**Table 1:** Ag content in roots and leaves after 7 days of tobacco plants exposure to 25, 50, and 100 µM AgNP–PVP, AgNP–CTAB, and AgNO_3_, applied either alone or in combination with 125, 250, and 500 µM cysteine (cys), respectively.

Plant Material	Treatments	Concentrations (µM)	Ag Content (μg g^−1^ DW)		
			AgNP–PVP	AgNP–CTAB	AgNO_3_
Root	Control	0	0.000 ± 0.000 ^c^	0.000 ± 0.000 ^d^	0.000 ± 0.000 ^d^
		25	3.601 ± 0.145 ^b,*B*^	5.348 ± 0.246 ^c,*A*^	2.712 ± 0.152 ^c,C^
	AgNP/AgNO_3_	50	14.833 ± 0.708 ^a,*A*^	8.885 ± 0.320 ^b,*B*^	3.833 ± 0.171 ^b,*C*^
		100	16.261 ± 0.283 ^a,*B*^	18.976 ± 0.635 ^a,*A*^	10.745 ± 0.038 ^a,*C*^
		25 + 125	1.847 ± 0.082 *, #	3.943 ± 0.184 *, #	0.788 ± 0.049 *, #
	AgNP/AgNO_3_ + cys	50 + 250	7.937 ± 0.342 *, #	6.386 ± 0.248 *, #	0.909 ± 0.055 *, #
		100 + 500	11.357 ± 0.646 *, #	12.678 ± 0.143 *, #	2.351 ± 0.042 *, #
Leaf	Control	0	0.000 ± 0.000 ^c^	0.000 ± 0.000 ^d^	0.000 ± 0.000 ^d^
		25	0.637 ± 0.101 ^c,*B*^	2.107 ± 0.509 ^c,*A*^	0.906 ± 0.037 ^c,*AB*^
	AgNP/AgNO_3_	50	5.253 ± 0.228 ^b,*A*^	3.399 ± 0.206 ^b,*B*^	1.402 ± 0.0504 ^b,*C*^
		100	7.437 ± 0.235 ^a,*B*^	8.936 ± 0.295 ^a*,A*^	5.298 ± 0.234 ^a,*C*^
		25 + 125	0.160 ± 0.509 *	0.902 ± 0.202	0.114 ± 0.005 *, #
	AgNP/AgNO_3_ + cys	50 + 250	1.041 ± 0.206 *, #	2.038 ± 0.138 *, #	0.290 ± 0.013 *, #
		100 + 500	3.376 ± 0.295 *, #	6.694 ± 0.658 *, #	1.155 ± 0.088 *, #

Values are the means ± standard error of three different experiments, each with three replicates. If values are marked with different letters, the treatments are significantly different at *p* ≤ 0.05 (a two-way ANOVA followed by Duncan’s post hoc test); small letters mark the differences among different concentrations of the same treatment type as well as control; capital letters mark the differences among different treatment types of the same concentration; asterisk (*) denotes significant differences among treatments with and without cysteine of the corresponding concentration, while hash sign (#) denotes significant difference between each treatment with cysteine and control. In control, Ag content was below the limit of quantification (LOQ < 0.1 µg g^-1^). DW—dry weight.

**Table 2:** Contents of reactive oxygen species (ROS), malondialdehyde (MDA), protein carbonyls, proline, reduced glutathione (GSH), and % tail DNA in roots of tobacco plants 7 days after exposure to 25, 50, and 100 μM AgNP–PVP, AgNP–CTAB, and AgNO_3_, applied either alone or in combination with 125, 250, and 500 µM cysteine (cys). FW—fresh weight.

	Conc. (µM)	ROS (% of Control)	MDA (nmol g^−1^ FW)	Protein Carbonyl (µmol mg^−1^ _proteins_)	% Tail DNA	Proline (µmol g^−1^ FW)	GSH (µmol g^−1^ FW)
Control	0	100 ± 6.11 ^b^	126.62 ± 2.95 ^ab^	0.025 ± 0.003 ^a^	4.70 ± 0.01 ^ab^	11.28 ± 1.64 ^a^	3.89 ± 0.13 ^a^
AgNP–PVP	25	180.45 ± 25.59 ^a,*A*^	139.78 ± 5.02 ^a,*A*^	0.029 ± 0.002 ^a,*A*^	3.80 ± 0.33 ^b,*A*^	9.04 ± 0.83 ^a,*A*^	3.54 ± 0.29 ^a,*A*^
	50	142.09 ± 14.86 ^ab,*A*^	125.03 ± 3.92 ^b,*A*^	0.032 ± 0.003 ^a,*A*^	4.10 ± 0.40 ^ab,*A*^	8.88 ± 0.79 ^a,*AB*^	3.02 ± 0.19 ^a,*A*^
	100	145.50 ± 6.92 ^ab,*A*^	106.97 ± 4.73 ^c,*B*^	0.029 ± 0.002 ^a,*A*^	5.18 ± 0.49 ^a,*A*^	8.21 ± 0.95 ^a,*A*^	4.04 ± 0.46 ^a,*A*^
AgNP–PVP + cys	25 + 125	114.61 ± 14.06 *	118.18 ± 3.71 *	0.030 ± 0.003	5.74 ± 0.43 *	9.68 ± 1.11	4.32 ± 0.36
	50 + 250	134.45 ± 12.64	123.44 ± 1.81	0.029 ± 0.003	5.03 ± 0.45	10.18 ± 0.86	4.93 ± 0.45 *, #
	100 + 500	150.30 ± 13.68 #	114.38 ± 8.47	0.030 ± 0.002	5.48 ± 0.48	11.58 ± 1.68	4.53 ± 0.53
Control	0	100 ± 4.39 ^a^	126.62 ± 2.76 ^a^	0.025 ± 0.002 ^a^	4.70 ± 0.01 ^a^	11.28 ± 1.79 ^a^	3.89 ± 0.12 ^a^
AgNP–CTAB	25	125.78 ± 14.46 ^a,*A*^	139.94 ± 9.27 ^a,*A*^	0.023 ± 0.003 ^a,*A*^	4.65 ± 0.46 ^a,*A*^	7.81 ± 0.90 ^a,*A*^	3.94 ± 0.37 ^a,*A*^
	50	104.24 ± 6.33 ^a*,AB*^	121.41 ± 4.71 ^a,*A*^	0.022 ± 0.003 ^a,*B*^	4.52 ± 0.43 ^a,*A*^	11.39 ± 1.02 ^a,*A*^	3.36 ± 0.38 ^ab,*A*^
	100	92.97 ± 8.66 ^a,*B*^	133.40 ± 7.21 ^a,*A*^	0.023 ± 0.002 ^a,*AB*^	2.84 ± 0.34 ^b,*B*^	9.15 ± 0.98 ^a,*A*^	2.96 ± 0.13 ^b,*B*^
AgNP–CTAB + cys	25 + 125	127.12 ± 12.23	119.18 ± 3.13 *	0.022 ± 0.002	4.51 ± 0.50	9.61 ± 0.94	3.95 ± 0.49
	50 + 250	102.31 ± 4.70	116.63 ± 6.86	0.019 ± 0.001 #	4.01 ± 0.38	9.16 ± 0.87	3.67 ± 0.18
	100 + 500	108.46 ± 14.83	115.12 ± 9.37	0.022 ± 0.001	4.18 ± 0.42 *	11.18 ± 1.25	3.97 ± 0.44 *
Control	0	100 ± 3.91 ^b^	126.62 ± 2.48 ^a^	0.025 ± 0.002 ^a^	4.70 ± 0.0.01 ^ab^	11.28 ± 1.09 ^a^	3.89 ± 0.10 ^a^
AgNO_3_	25	169.16 ± 11.32 ^a,*A*^	108.02 ± 3.02 ^b,*B*^	0.027 ± 0.003 ^a,*A*^	3.57 ± 0.33 ^b,*A*^	9.11 ± 1.80 ^ab,*A*^	3.47 ± 0.17 ^ab,*A*^
	50	86.76 ± 9.80 ^b,*B*^	115.89 ± 5.39 ^b,*A*^	0.022 ± 0.001 ^a,*B*^	3.81 ± 0.39 ^b,*A*^	6.64 ± 0.30 ^b,*B*^	3.31 ± 0.16 ^b,*A*^
	100	76.95 ± 5.15 ^b,*B*^	95.39 ± 2.17 ^c,*B*^	0.022 ± 0.001 ^a,*B*^	5.80 ± 0.46 ^a,*A*^	11.11 ± 0.90 ^a,*A*^	3.41 ± 0.10 ^b,*AB*^
AgNO_3_ + cys	25 + 125	87.67 ± 1.95 *	101.58 ± 2.18 #	0.039 ± 0.001 *, #	5.35 ± 0.46 *	6.26 ± 0.63 #	4.21 ± 0.43
	50 + 250	83.84 ± 8.03	102.65 ± 4.42 *, #	0.030 ± 0.002 *	5.56 ± 0.44 *	10.41 ± 0.52 *	5.79 ± 0.32 *, #
	100 + 500	69.84 ± 1.17	113.19 ± 6.69 *, #	0.043 ± 0.002 *, #	5.40 ± 0.43	12.07 ± 1.21	4.25 ± 0.36 *

Values are the means ± SE of three biological replicates, each with six technical replicas. If values are marked with different letters, the treatments are significantly different at *p* ≤ 0.05 (a one way ANOVA followed by Duncan’s post hoc test); small letters mark the differences among different concentrations of the same treatment type as well as control; capital letters mark the differences among different treatment types of the same concentration; asterisk (*) denotes significant differences among treatments with and without cysteine of the corresponding concentration, while hash sign (#) denotes significant difference between each treatment with cysteine and control.

**Table 3:** Contents of reactive oxygen species (ROS), malondialdehyde (MDA), protein carbonyls, proline, reduced glutathione (GSH), and % tail DNA in leaves of tobacco plants 7 days after exposure to 25, 50, and 100 μM AgNP–PVP, AgNP–CTAB, and AgNO_3_, applied either alone or in combination with 125, 250, and 500 µM cysteine (cys). FW—fresh weight.

	Conc. (µM)	ROS (% of Control)	MDA (nmol g^−1^ FW)	Protein Carbonyls (µmol mg^−1^ _proteins_)	% Tail DNA	Proline (µmol g^−1^ FW)	GSH (µmol g^−1^ FW)
Control	0	100 ± 11.18 ^ab^	157.51 ± 4.13 ^a^	0.022 ± 0.002 ^a^	4.20 ± 0.165 ^b^	10.40 ± 0.49 ^b^	3.59 ± 0.23 ^c^
AgNP–PVP	25	72.15 ± 12.82 ^b,*A*^	180.07 ± 7.20 ^a,*A*^	0.023 ± 0.001 ^a,*B*^	5.46 ± 0.49 ^a,*A*^	16.61 ± 1.75 ^a,*A*^	5.30 ± 0.35 ^ab,*B*^
	50	100.45 ± 6.70 ^ab,*A*^	165.19 ± 11.88 ^a,*A*^	0.019 ± 0.001 ^a,*B*^	3.87 ± 0.36 ^b,*A*^	17.08 ± 1.91 ^a,*A*^	6.24 ± 0.55 ^a,*A*^
	100	119.08 ± 14.12 ^a,*A*^	161.47 ± 7.46 ^a,*A*^	0.019 ± 0.002 ^a,*B*^	4.39 ± 0.39 ^ab,*A*^	16.83 ± 2.18 ^a,*A*^	4.21 ± 0.52 ^bc,*C*^
AgNP–PVP + cys	25 + 125	108.68 ± 5.77 *	148.71 ± 6.72 *	0.024 ± 0.001	5.70 ± 0.49 #	11.32 ± 0.99 *	4.25 ± 0.39 *
	50 + 250	78.18 ± 8.88	143.68 ± 6.32	0.017 ± 0.001	6.65 ± 0.54 *, #	10.72 ± 0.75 *	3.86 ± 0.27 *
	100 + 500	111.38 ± 9.32	152.23 ± 7.16	0.017 ± 0.002	5.03 ± 0.45	12.06 ± 0.92 *	4.03 ± 0.19
Control	0	100 ± 11.14 ^a^	157.51 ± 2.55 ^b^	0.022 ± 0.002 ^b^	4.20 ± 0.16 ^a^	10.40 ± 0.55 ^b^	3.59 ± 0.17 ^c^
AgNP–CTAB	25	103.16 ± 17.26 ^a,*A*^	170.29 ± 6.47 ^ab,*A*^	0.033 ± 0.001 ^a,*A*^	5.12 ± 0.48 ^a,*A*^	11.32 ± 1.02 ^b,*B*^	6.10 ± 0.93 ^b,*AB*^
	50	98.81 ± 12.58 ^a,*A*^	195.36 ± 6.88 ^a,*A*^	0.033 ± 0.001 ^a,A^	4.71 ± 0.49 ^a,*A*^	10.53 ± 0.73 ^b,*B*^	7.66 ± 0.86 ^ab,*A*^
	100	111.47 ± 13.49 ^a,*A*^	155.19 ± 4.77 ^b,*A*^	0.033 ± 0.002 ^a,*A*^	4.27 ± 0.58 ^a,*A*^	16.85 ± 2.48 ^a,*A*^	9.08 ± 0.51 ^a,*A*^
AgNP–CTAB + cys	25 + 125	109.70 ± 8.47	187.36 ± 7.59 *^,^ #	0.024 ± 0.003 *	6.19 ± 0.63 #	9.33 ± 0.67	5.38 ± 0.33 #
	50 + 250	67.13 ± 5.20	177.63 ± 7.45	0.027 ± 0.003	4.09 ± 0.38	11.20 ± 0.55	5.54 ± 0.42 *, #
	100 + 500	95.66 ± 7.27	153.76 ± 7.31	0.028 ± 0.003	4.72 ± 0.38	12.53 ± 0.91 *	6.43 ± 0.56 *, #
Control	0	100 ± 3.96 ^b^	157.51 ± 5.19 ^a^	0.022 ± 0.002 ^a^	4.20 ± 0.16 ^a^	10.40 ± 0.62 ^b^	3.59 ± 0.25 ^c^
AgNO_3_	25	70.61 ± 4.90 ^c,*A*^	158.75 ± 5.21 ^a,*A*^	0.023 ± 0.001 ^a,*B*^	3.15 ± 0.35 ^a,*B*^	10.17 ± 0.54 ^b,*B*^	7.76 ± 0.65 ^a,*A*^
	50	77.20 ± 8.15 ^c,*A*^	171.73 ± 8.43 ^a,*A*^	0.020 ± 0.002 ^a,*B*^	4.07 ± 0.37 ^a,*A*^	13.31 ± 1.33 ^b,*AB*^	5.88 ± 0.42 ^b,*A*^
	100	123.47 ± 4.46 ^a,*A*^	173.78 ± 9.50 ^a,*A*^	0.022 ± 0.002 ^a*,B*^	3.87 ± 0.42 ^a,*A*^	20.24 ± 2.4 ^a,*A*^	6.65 ± 0.60 ^ab,*B*^
AgNO_3_ + cys	25 + 125	126.92 ± 8.21 *, #	155.05 ± 5.28	0.019 ± 0.002	5.07 ± 0.42 *	10.90 ± 0.87	6.80 ± 0.24 #
	50 + 250	89.65 ± 17.16	157.24 ± 4.31	0.019 ± 0.002	5.51 ± 0.52 *, #	10.99 ± 0.57	3.35 ± 0.36 *
	100 + 500	104.99 ± 16.55	156.48 ± 8.86	0.022 ± 0.001	6.56 ± 0.46 *, #	12.65 ± 1.26 *	3.84 ± 0.54 *

Values are the means ± SE of three biological replicates, each with six technical replicas. If values are marked with different letters, the treatments are significantly different at *p* ≤ 0.05 (a one-way ANOVA followed by Duncan’s post hoc test); small letters mark the differences among different concentrations of the same treatment type as well as control; capital letters mark the differences among different treatment types of the same concentration; asterisk (*) denotes significant differences among treatments with and without cysteine of the corresponding concentration, while hash sign (#) denotes significant difference between each treatment with cysteine and control.

## Data Availability

Not applicable.

## Supplementary Materials

The following supporting information can be downloaded at: https://www.mdpi.com/article/10.3390/plants11182402/s1, Figure S1: AgNP localization in root cells of tobacco plant treated with 100 μM AgNP–PVP. TEM microphotograph of root cell with AgNPs in the cytoplasm of the epidermal cell (A), silver elemental map (B), and energy-dispersive X-ray spectrum (C). CW—cell wall; Cyt—cytoplasm; Figure S2: AgNP localization in root cells of tobacco plant treated with 100 μM AgNP–CTAB. TEM microphotograph of root cell with AgNPs in the cell cytoplasm (A), enlarged part of cytoplasm with AgNPs (B), silver elemental map (C), and energy-dispersive X-ray spectrum (D). CW—cell wall; Cyt—cytoplasm; V—vacuole; Table S1: Physicochemical characteristics of AgNP–PVP and AgNP–CTAB in stock solutions by means of hydrodynamic diameter (dH) in nm obtained from size distributions by volume, ζ potential values in mV, surface plasmon resonance (SPR), and percentage of ionic Ag^+^.
