# Supplementary material for: Silver Nanoparticle Effects on Antioxidant Response in Tobacco Are Modulated by Surface Coating
Source: Plants (Basel). 2022 Sep 15;11(18):2402. doi: 10.3390/plants11182402 (PMC9504990; doi:10.3390/plants11182402)
Supplement: Supplementary file 1 [file plants-11-02402-s001.zip › Figure S1. AgNP-PVP localization.pdf]

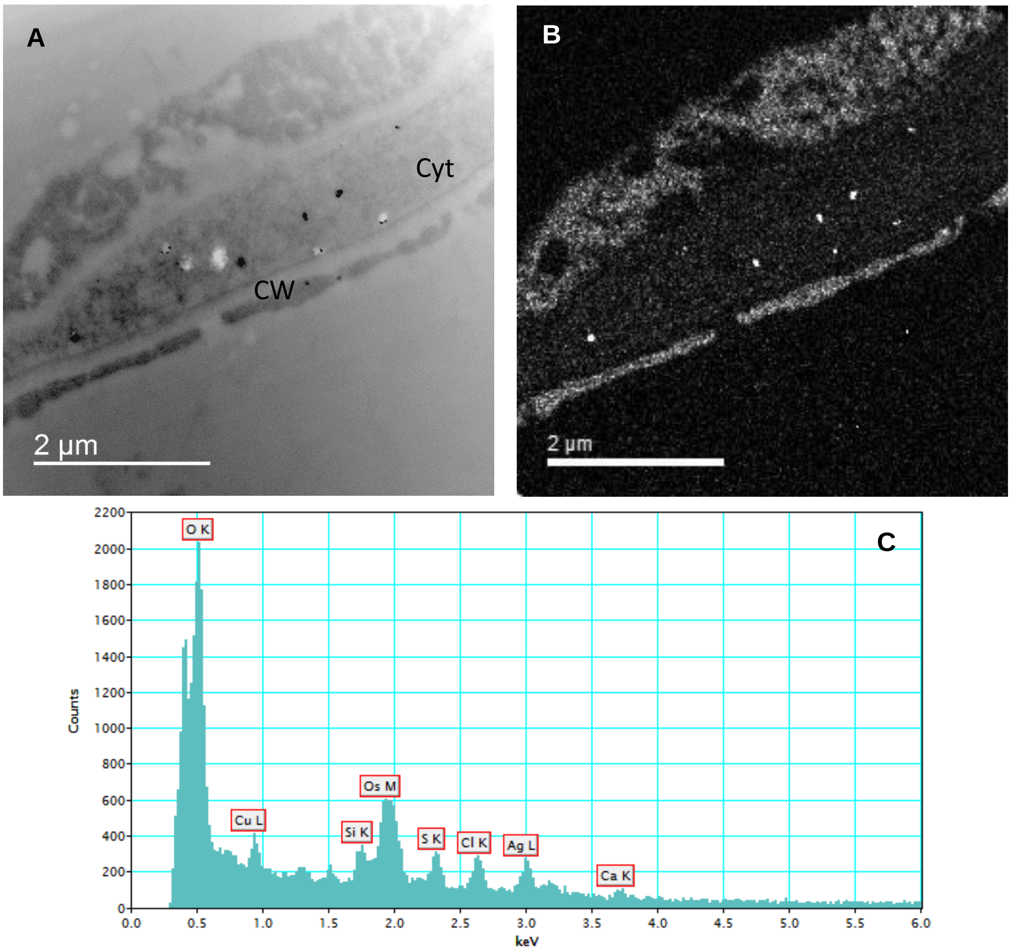

Figure S1. AgNP localization in root cells of tobacco plant treated with 100 μM AgNP-PVP. TEM microphotograph of root cell with AgNPs in the cytoplasm of the epidermal cell (**A**), silver elemental map (**B**), and energy-dispersive X-ray spectrum (**C**). CW - cell wall; Cyt - cytoplasm
