# Supplementary material for: Silver Nanoparticle Effects on Antioxidant Response in Tobacco Are Modulated by Surface Coating
Source: Plants (Basel). 2022 Sep 15;11(18):2402. doi: 10.3390/plants11182402 (PMC9504990; doi:10.3390/plants11182402)
Supplement: Supplementary file 1 [file plants-11-02402-s001.zip › Table S1. AgNPs.pdf]

Table S1. Physico-chemical characteristics of AgNP-PVP and AgNP-CTAB in stock solutions by means of hydrodynamic diameter (d_H) in nm obtained from size distributions by volume, ζ potential values in mV, surface plasmon resonance (SPR) and percentage of ionic Ag^+.

**Characteristics**		**AgNP-PVP**	**AgNP-CTAB**
Size peak I	d_H, nm	57.65 ± 16.84	28.19 ± 10.19
	mean volume, %	100%	18.1%
Size peak II	d_H, nm		56.36 ± 11.56
	mean volume, %		23.9%
Size peak III	d_H, nm		162.25 ± 86.14
	mean volume, %		58.0%
ζ potential, mV		-4.24 ± 2.57	44.67 ± 3.36
SPR peak, nm		465	410
Ag^+, %		0.3	0.5
Working stock concentrations, mM		10.2	10.5
